# Signatures of Multi-Omics Reveal Distinct Tumor Immune Microenvironment Contributing to Immunotherapy in Lung Adenocarcinoma

**DOI:** 10.3389/fimmu.2021.723172

**Published:** 2021-09-03

**Authors:** Ziqi Huang, Baihui Li, Yan Guo, Lei Wu, Fan Kou, Lili Yang

**Affiliations:** ^1^Department of Immunology, Tianjin Medical University Cancer Institute and Hospital, Tianjin, China; ^2^National Clinical Research Center for Cancer, Tianjin, China; ^3^Key Laboratory of Cancer Prevention and Therapy, Tianjin, China; ^4^Key Laboratory of Cancer Immunology and Biotherapy, Tianjin, China

**Keywords:** lung cancer, MET, CNV, multi-omic analysis, tumor immune microenvironment, PD-L1, immunotherapy

## Abstract

**Background:**

Lung adenocarcinoma (LUAD) contains a variety of genomic and epigenomic abnormalities; the effective tumor markers related to these abnormalities need to be further explored.

**Methods:**

Clustering analysis was performed based on DNA methylation (MET), DNA copy number variation (CNV), and mRNA expression data, and the differences in survival and tumor immune microenvironment (TIME) between subtypes were compared. Further, we evaluated the signatures in terms of both prognostic value and immunological characteristics.

**Results:**

There was a positive correlation between MET and CNV in LUAD. Integrative analysis of multi-omics data from 443 samples determined molecular subtypes, iC1 and iC2. The fractions of CD8+ T cells and activated CD4+ T cells were higher, the fraction of Tregs was lower, and the expression level of programmed death-ligand 1 (PD-L1) was higher in iC2 with a poor prognosis showing a higher TIDE score. We selected *PTTG1*, *SLC2A1*, and *FAM83A* as signatures of molecular subtypes to build a prognostic risk model and divided patients into high-risk group and low-risk group representing poor prognosis and good prognosis, respectively, which were validated in 180 patients with LUAD. Further, the low-risk group with lower TIDE score had more infiltrating immune cells. In 100 patients with LUAD, the high-risk group with an immunosuppressive state had a higher expression of PD-L1 and lower counts of CD8+ T cells and dendritic cells.

**Conclusions:**

These findings demonstrated that combined multi-omics data could determine molecular subtypes with significant differences of prognosis and TIME in LUAD and suggested potent utility of the signatures to guide immunotherapy.

## Introduction

Lung cancer, the second largest cancer in the world, remains the leading cause of cancer death, with a 5-year survival rate of about 19% for the vast majority of patients in China (1, 2). Lung adenocarcinoma (LUAD) is the main histological subtype of lung cancer and shows distinct states at the transcriptome level and in the cell control network, with unique genetic drivers and different prognostic characteristics (3, 4). The correlation between classic driver oncogene mutations and the tumor immune microenvironment (TIME) according to the presence of programmed death-ligand 1 (PD-L1) and tumor-infiltrating lymphocytes (TILs) is close in LUAD (5, 6). The generation of novel drugs targeting key disease driver mutations has created optimism for the treatment of LUAD (7). Therefore, it is of great clinical significance to identify effective tumor markers and study their role in the development of LUAD for early treatment.

LUAD contains a variety of genomic and epigenomic abnormalities. DNA methylation (MET) is a form of epigenetic modification; MET may negatively influence transcription by impeding the induction of genes needed for epigenetic reprogramming (8, 9). Recently, it has been reported that the transcription level of immune infiltration genes such as cytotoxic Tlymphocyte-associated protein 4 (CTLA4) and granzyme A (GZMA) seems to be highly correlated with methylation of specific CpG markers in the promoter region, suggesting a connection between methylation and immune cell infiltration (10). Another study showed that specific p53 mutants are related to the immune subtype of ovarian cancer (11). DNA copy number variations (CNV) are somatic gene changes that drive cancer (12). Large amounts of CNV are present in lung cancer and breast cancer, the *BCL2* family of apoptosis regulators and the NF-κB pathway are enriched among these regions of focal CNV, and cancer cells depend on the dysregulated expression of *BCL2L1* for survival (13). The CNV may be enriched for immune system genes and include genes that may contribute to the recruitment of immune cells (14).

Advances in high-throughput experimental methods and the development of joint clustering algorithms make it possible to cluster multi-omics data to reveal more system-level insights (15). Recently, integrative multi-omics analysis based on somatic mutation, copy number aberration, and gene expression profile has brought a new perspective to the TIME in triple-negative breast cancer (TNBC) (16, 17). It is necessary to investigate the TIME of LUAD with significantly altered T cell compartments and PD-L1-associated immunoediting from this integrative perspective (18, 19).

At present, the effective tumor markers related to abnormalities of MET and CNV in LUAD need to be further explored. In this study, we not only identified subtypes with different outcomes, different fractions of immune cells, and different expression levels of immune checkpoints but also found out that effective signatures could indicate these differences in outcome and TIME. Finally, the prognostic value and immunological characteristics of the signatures were validated in samples of patients with LUAD.

## Materials and Methods

### TCGA Data Download and Preprocessing

MET data, CNV data, and single-nucleotide polymorphism (SNP) data of LUAD samples were downloaded from the TCGA project (http://firebrowse.org/); in addition, mRNA expression data and clinical information of LUAD samples were downloaded (https://portal.gdc.cancer.gov/). Mutation allelic fraction (MAF) data were downloaded for calculation of tumor mutational burden (TMB) by R-packet “Maftools.” A total of 443 LUAD samples of all sets of data were used in subsequent analyses.

The sites with missing values were deleted, and the probes from the upstream 1,500 bp of transcription start sites (TSS) to the downstream gene body region were matched to the corresponding genes in MET data. The region with less than five probes was filtered out in the CNV data. The mRNA expression data preprocessing method deleted the genes whose expression value was greater than 0 in less than 5% samples and deleted silencing mutation and intron mutation in SNP data.

### Data Processing

The Genomic Identification of Significant Targets in Cancer (GISTIC) method was used to detect the region of common copy number variations in all samples, including the horizontal copy number variation of the chromosome arm and the minimum common region among samples. The parameters of the GISTIC method were set as Q ≤ 0.05 as the significance criterion of change. When determining the peak interval, the confidence level is 0.95. When analyzing the horizontal variation of the chromosome arm, the region larger than the length of the chromosome arm which is 0.98 was used as the standard, Development of the Broad Institute online analysis tool GenePattern (https://cloud.genepattern.org/gp/pages/index.jsf) was analyzed in the corresponding MutSigCV module (20). Based on GISTIC, the CNV information of each sample was defined: β > 0.3 was defined as copy number amplification (Gain), and β < -0.3 was defined as copy number deficiency (Loss); the methylation level of the samples was also defined: β > 0.6 was defined as high methylation (MetHyper), and β < 0.4 was defined as low methylation (MetHypo).

The differentially expressed gene (DEG) analysis of mRNA data was performed using R-packet “Limma” (21). The screening threshold criteria were |log2—a fold change (FC)| > 1.0 and FDR < 0.05.

### Clustering Analysis

First, Pearson correlation coefficients between MET/CNV and mRNA expression data were calculated. The correlation coefficients were converted to Z value according to the formula In(1+r)/(1-r). In the correlation coefficient test, the gene with p < 0.05 is considered to be the MET correlation (METcor) gene or CNV correlation (CNVcor) gene. In order to filter out unnecessary genes, we identified DEGs related to prognosis between 443 tumor samples and 59 healthy samples using univariate Cox regression analysis. Finally, 343 DEGs were included in the METcor gene set and the CNVcor gene set for subsequent analysis.

Second, we used the R-packet “non-negative matrix factorization” (NMF) analysis to identify the clusters of tumors by extracting the characteristics of the samples. METcor/CNVcor clusters were clustered according to the mRNA expression data of the METcor/CNVcor gene set; with the standard “Brunet” method, 50 iterations were employed and cluster number *k* was set at 2–5, which were sufficient to achieve the optimal cluster *k* = 2 on the basis of cophenetic, dispersion, and silhouette.

### Kaplan–Meier Survival Analysis

Overall survival (OS) is the period between surgical resection and death or the last follow-up. Disease-free survival (DFS) was the time to relapse or death from any cause. Progression-free survival (PFS) was the time between surgical resection and progression. Survival information was obtained from TCGA and Tianjin Medical University Cancer Institute and Hospital. Kaplan–Meier curves were used to assess the differences in survival with the log-rank test. We calculated sensitivity and specificity by the ROC method to determine the optimal cutoff values for the continuous variables. According to the optimal cutoff value, we divided the cohort into two subgroups.

### Evaluation of TIME

The immune cell infiltration features of TIME between TCGA clusters were analyzed by estimating relative subsets of RNA transcripts (CIBERSORT), and we compared the fractions of 22 immune cell types (22). The ESTIMATED algorithms were used to evaluate immune scores, stromal scores, and tumor purity for each sample. The online tool Tumor Immune Dysfunction and Exclusion (TIDE) (http://tide.dfci.harvard.edu) was used to predict the immunotherapeutic responses of each sample based on the transcriptome profiles.

### Integrative Analysis

Integrative analysis based on multi-omics data was performed by R-packet “iCluster” (23). “iCluster” is designed to type tumors based on the NMF method using a combination of MET data from the METcor gene set, CNV data from the CNVcor gene set, and mRNA expression data from both gene sets. Subsequently, 50 iterations and 10 lambda sample points between 0 and 1 were used for optimal lambda value screening to identify the optimal MET, CNV, and mRNA expression data weight values (lambda values). Considering the number of clusters identified by the METcor/CNVcor gene set, we chose 2 as the number of clustering *k*.

### Establishment of a Prognostic Risk Model

We selected signatures in three steps. First, we performed univariate Cox regression analysis then continued with the least absolute correlation and selection operator (Lasso) regression analysis. Finally, the results were followed by multivariate Cox regression analysis. According to the expression of signatures and the prognosis information, we build the risk model by the multivariate Cox method and calculated the risk score of each patient using the R-packet “survival.”

### Validation in the External Data Set

We download the LUAD dataset GSE31210 from the GEO database, including 226 samples with complete follow-up data. Then, we calculated the risk score according to the formula (risk score = *PTTG1* expression level* 0.026624+ *SLC2A1* expression level* 0.005851+ *FAM83A* expression level* 0.006776) and divided the cohort to analysis survival.

### Tumor Tissue Samples

A total of 180 LUAD patients were enrolled in this study (Tianjin Medical University Cancer Institute and Hospital, China), and informed consent was obtained from all patients. The lung cancer stages were categorized according to the International Association for the Study of Lung Cancer TNM staging system. The use of patient information and tissues was approved by the Ethics Committee of the Tianjin Medical University Cancer Institute and Hospital.

### RT-qPCR

cDNA was synthesized by PrimeScript™ RT Master Mix (TaKaRa). Quantitative RT-PCR (RT-qPCR) was performed with primers of *PTTG1*, *SLC2A1*, and *FAM83A* and analyzed by the comparative Ct value (2-ΔΔCt). Primer sequences of *PTTG1* are forward GACTTTGAGAGTTTTGACCTGC and reverse GAGACTGCAACAGATTGGATTC. Primer sequences of *SLC2A1* are forward GATGAAGGAAGAGAGTCGGCAGATG and reverse CAGCACCACAGCGATGAGGATG. Primer sequences of *FAM83A* are forward GCTGACTTTAGTGACAACGAGA and reverse CTCCACCGAGGACAAGAAG. The RNA samples were derived from the Tianjin cohort; we mixed 10 adjacent samples as reference and detected the relative expression level in 41 tumor samples.

### Immunohistochemistry

We selected paraffin-embedded tissue microarray (TMA) for immunohistochemistry (IHC). IHC was performed as previously described (24). The appropriate primary antibody was added to cover the tissue, and slides were incubated at 4°C. Finally, the sections were stained with DAB Substrate Kit. The images were captured by DP Manager software (×400 magnification, OLYMPUS), and the H-score was evaluated by two independent pathologists.

Primary antibodies include the anti-*PTTG1* antibody (PA5-29399, Invitrogen, USA, 1:400), anti-*SLC2A1* antibody (MA5-11315, Invitrogen, USA, 1:200), anti-*FAM83A* antibody (SAB2108978, Sigma, USA, 1:200), anti-CD4 antibody (ab133616, Abcam, USA, 1:200), anti-CD8 antibody (SP16, Invitrogen, USA, 1:200), anti-CD20 antibody (EP459Y, Abcam, USA, 1:200), anti-CD68 antibody (KP1, Invitrogen, USA, 1:100), and anti-CD11c antibody (EP1347Y, Abcam, USA, 1:200).

### Statistical Analysis

The χ^2^ goodness-of-fit test or Fisher’s exact test was used to analyze categorical data. The t test or one-way ANOVA was used to analyze continuous data. All analyses were performed using IBM SPSS (version 21) and GraphPad Prism 8.3.0 software. p < 0.05 was considered to indicate a statistically significant difference.

## Results

### Different Outcomes and Different Patterns of Immune Cell Infiltration and Immune Checkpoints Between METcor Clusters

**Figure 1:**
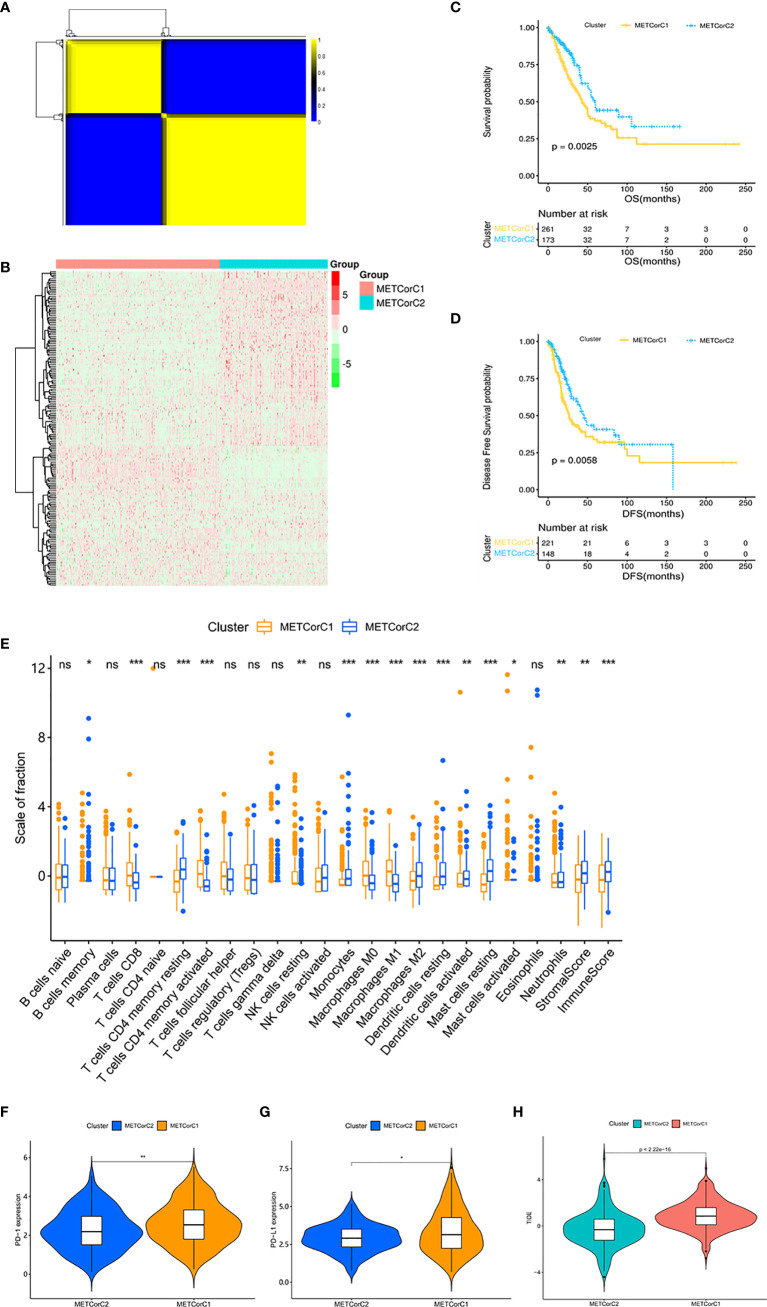
Different outcomes and different patterns of immune cell infiltration and immune checkpoints between METcor clusters. **(A)** The clustering results of NMF. Yellow represents high correlation, and the number of yellow squares represents the number of clusters. **(B)** Heat map of the beta values related to the METcor gene set. **(C)** Survival analysis with the Kaplan–Meier curve showed that the OS rate in METCorC1 was significantly lower than that in METCorC2 (p = 0.0025); **(D)** the DFS rate in METCorC1 was significantly lower than that in METCorC2 (p = 0.0058). Significance was determined using the log-rank p test. **(E)** The fractions of 22 immune cell types, stromal score, and immune score in METCorC1 and METCorC2. **(F, G)** Expression level of **(F)** PD-1 and **(G)** PD-L1 in METCor clusters. **(H)** TIDE score in METcor clusters. *p < 0.05, **p < 0.01, and ***p < 0.001. ns, not statistically significant.

In order to facilitate the study of the effect of MET in LUAD, we conducted clustering analysis based on MET data at first. The correlation analysis between MET and mRNA expression calculated in 443 samples from the TCGA cohort (**Supplementary Table 1**) showed that the MET level of 7,668 genes was correlated with mRNA expression (p < 0.05). We further identified the METcor gene set (n = 180) through filtering of DEGs related to prognosis (**Supplementary Table 2**). With the R-packet “NMF”, we set the cluster number *k* as 2–5 (**Supplementary Figure 1A**), carried out 50 iterations, and determined the optimal cluster number *k* as 2 (**Figure 1A**). The principal component analysis (PCA) showed that the samples were separated into two METcor clusters (**Supplementary Figure 1B**), METCorC1 (n = 267) and METCorC2 (n = 176). The beta values related to the METcor gene set were shown (**Figure 1B**). In addition, METCorC1 showed worse prognosis in OS (p = 0.0025) and DFS (p = 0.0058) than METCorC2 (**Figures 1C, D**).

Next, we evaluated the level of immune cell infiltration in the METcor clusters. In METCorC1, the total immune score, the stromal score, and the fraction of DC were lower (p < 0.01; **Figure 1E**). In addition, the fractions of CD8+ T cells and activated CD4+ T cells were higher (p < 0.001), and the fraction of resting CD4+ T cells was lower (p < 0.001) in METCorC1, while there was no difference in Tregs between the two clusters. For macrophage, the fractions of M0 and M1 were higher (p < 0.001) and the fraction of M2 was lower (p < 0.001) in METCorC1. In order to further investigate the TIME of the METcor clusters, we detected the immune checkpoints (**Figures 1F, G** and **Supplementary Figures 1C–F**) targeted in clinical immunotherapy of lung cancer. The results showed that the expression level of programmed death 1 (PD-1), PD-L1, indoleamine 2,3-dioxygenase 1 (IDO), and lymphocyte-activating 3 (LAG3) in METCorC1 was higher than in METCorC2 (p < 0.05), suggesting the deep contribution of tumor immune evasion in METCorC1. The high score of TIDE analysis in METCorC1 elucidated the worse outcome with high level of infiltration by cytotoxic T cells (p < 0.001; **Figure 1H**). These findings suggested that the immunosuppressive status caused by dysfunction of T cells and the high expression of immune checkpoints may have an important impact on clinical outcomes.

In the previous study, we used the same method to identify the CNVcor gene set (n = 160) and determine the CNVcor clusters (25). Also, we assessed the level of immune cell infiltration and the expression of immune checkpoints in the CNVcor clusters. Similarly, the fraction of DC was lower (p < 0.05), and both the fractions of CD8+ T cells and activated CD4+ T cells were higher (p < 0.001) in CNVCorC1 with worse prognosis (**Supplementary Figure 2A**). Moreover, there was a slight difference in PD-1, IDO, LAG3, and hepatitis A virus cellular receptor 2 (TIM3) expression between the two clusters (p < 0.05), but no difference in PD-L1 and (CTLA4; **Supplementary Figures 2B–G**). Consistent with the results of METcor clusters, T cells of CNVCorC1 with poor outcome tend to be in a dysfunctional state (p < 0.001; **Supplementary Figure 2H**).

### Multi-Omics Data Identified Molecular Subtypes With Distinct TIME

**Figure 2:**
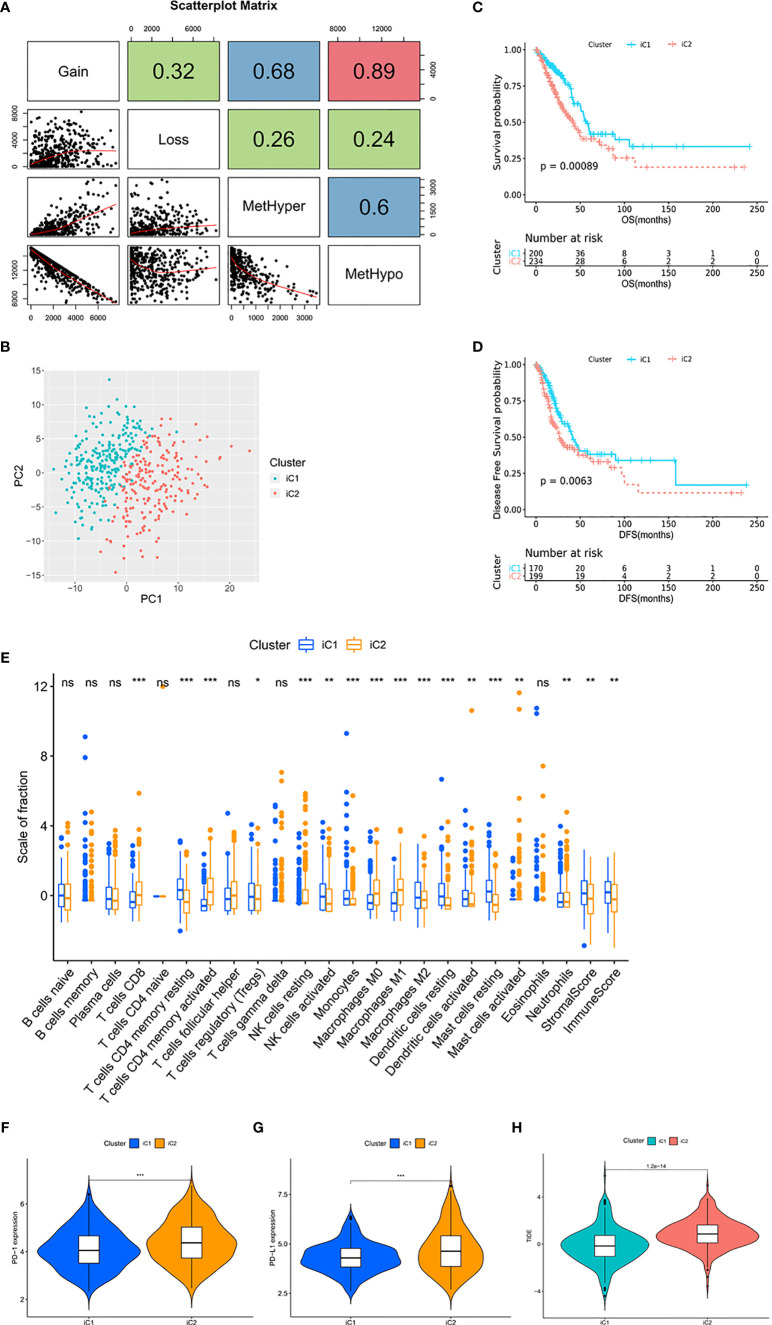
Multi-omics data identified molecular subtypes with distinct TIME. **(A)** Scatter distribution of MetHyper/MetHypo/gain/loss frequencies from samples. The number in the box represents the correlation coefficient of the corresponding position; each dot represents a sample. The Spearman method was used to calculate the correlation; p < 0.001. **(B)** The PCA of the two molecular subtypes. The green dots represent the patients in iC1 (n = 203). The red dots represent the patients in iC2 (n = 240). **(C)** Survival analysis with the Kaplan–Meier curve showed that the OS rate in iC1 was significantly higher than that in iC2 (p < 0.001); **(D)** the DFS rate in iC1 was significantly higher than that in iC2 (p = 0.0063). Significance was determined using the log-rank p test. **(E)** The fractions of 22 immune cell types, stromal score, and immune score in iC1 and iC2. **(F, G)** Expression level of **(F)** PD-1 and **(G)** PD-L1 in iC subtypes. **(H)** TIDE score in iC subtypes. *p < 0.05, **p < 0.01, and ***p < 0.001. ns, not statistically significant.

In the process of determining the METcor and CNVcor gene sets, we found that some genes were co-correlated with both MET and CNV, and there was overlap between the METcor clusters and CNVcor clusters (**Supplementary Figures 3A–C**). In order to explore the correlation between MET and CNV, we counted the number of MetHyper, MetHypo, Gain, and Loss from each sample, respectively (**Figure 2A** and **Supplementary Table 3**). Correlation analysis showed that Gain was positively correlated with both Loss (r = 0.32, p < 0.001) and MetHyper (r = 0.68, p < 0.001), and Loss was positively correlated with MetHyper in the meantime (r = 0.26, p < 0.001). Moreover, there was a strong negative correlation between Gain and MetHypo (r = -0.89, p < 0.001), and MetHypo was negatively correlated with both Loss (r = -0.24, p < 0.001) and MetHyper (r = -0.6, p < 0.001). The results suggest that LUAD patients with abnormal MET are more likely to be accompanied by CNV abnormalities. In addition, a recent study reported a similar phenomenon in ovarian cancer (26). The correlation might be related to the distribution of METcor genes and CNVcor genes on the chromosome, and the variations of copy number could make epigenetic modification more convenient (**Supplementary Table 4**). Such findings highlight the clinical need for multi-omics analysis of MET data and CNV data for early diagnosis and accurate prognosis predictions in LUAD.

In view of the positive correlation between MET and CNV, we combined the data of MET and CNV to perform integrative analysis and divided the TCGA cohort into two molecular subtypes (**Figure 2B**), iCluster 1 (iC1, n = 203) and iCluster 2 (iC2, n = 240). With overlaps between METcor/CNVcor clusters and iC subtypes, survival analysis showed that OS (p = 0.00089) and DFS (p = 0.0063) were significantly longer in iC1 compared with iC2 (**Figures 2C, D** and **Supplementary Figures 3D, E**). It was suggested that the integrative analysis based on gene profile, epigenetic profile, and mRNA expression profile is capable for predicting the prognosis in LUAD.

Next, we evaluated the level of immune cell infiltration in the molecular subtypes. In iC1, the total immune score, the stromal score, and the fractions of DC, activated NK cells were higher (p < 0.01; **Figure 2E**). In addition, the fractions of CD8+ T cells and activated CD4+ T cells were lower (p < 0.001), and the fraction of resting CD4+ T cells was higher (p < 0.001) in iC1, while there was a slight difference in Tregs between the two subtypes (p < 0.05). For macrophage, the fractions of M0 and M1 were lower (p < 0.001) and the fraction of M2 was higher (p < 0.001) in iC1. The expression level of PD-1, PD-L1, IDO, and LAG3 in iC2 was significantly higher than in iC1 (p < 0.001; **Figures 2F, G** and **Supplementary Figures 3F–I**), and the TIDE score was lower in iC1 compared with iC2 (p < 0.001; **Figure 2H**), suggesting the severely immunosuppressive TIME in iC2. In conclusion, the molecular subtypes showed significant differences in TIME, especially the differences in the expression of immune checkpoints, suggesting the potential value of this approach in guiding immunotherapy.

In addition, we used Fisher’s exact test and observed 1,704 significantly different genes in MET and 3,362 significantly different genes in CNV between iC1 and iC2 in order to identify molecular features of the two subtypes (**Supplementary Figures 4A, B**). Further, we investigated the CNV distribution of driver genes (**Supplementary Figure 4C**) and the SNP distribution of the top 15 significant genes (**Supplementary Figure 4D**). We found that most driver genes of iC1 were more likely to show a Loss status of CNV, and the *TP53* mutation rate (54%) was the highest in the cohort.

### Prognostic Risk Model Built by *PTTG1*, *SLC2A1*, and *FAM83A*

Then, we conducted the enrichment analysis based on 275 DEGs in the mRNA expression profile to explore the potential regulatory mechanism of heterogeneous progression between the two molecular subtypes (**Supplementary Figure 4E**). The Kyoto Encyclopedia of Genes and Genomes (KEGG) analysis showed that the DEGs were mainly concentrated in apoptosis, cell cycle, and P53 signaling pathways (**Supplementary Figure 4F**). The Gene Ontology (GO) analysis showed that the DEGs were enriched in terms of nuclear division, spindle, and microtubule binding (**Supplementary Figure 4G**). The results indicated that the differences of biological functions caused by these pathways between the two subtypes might contribute to heterogeneity.

**Figure 3:**
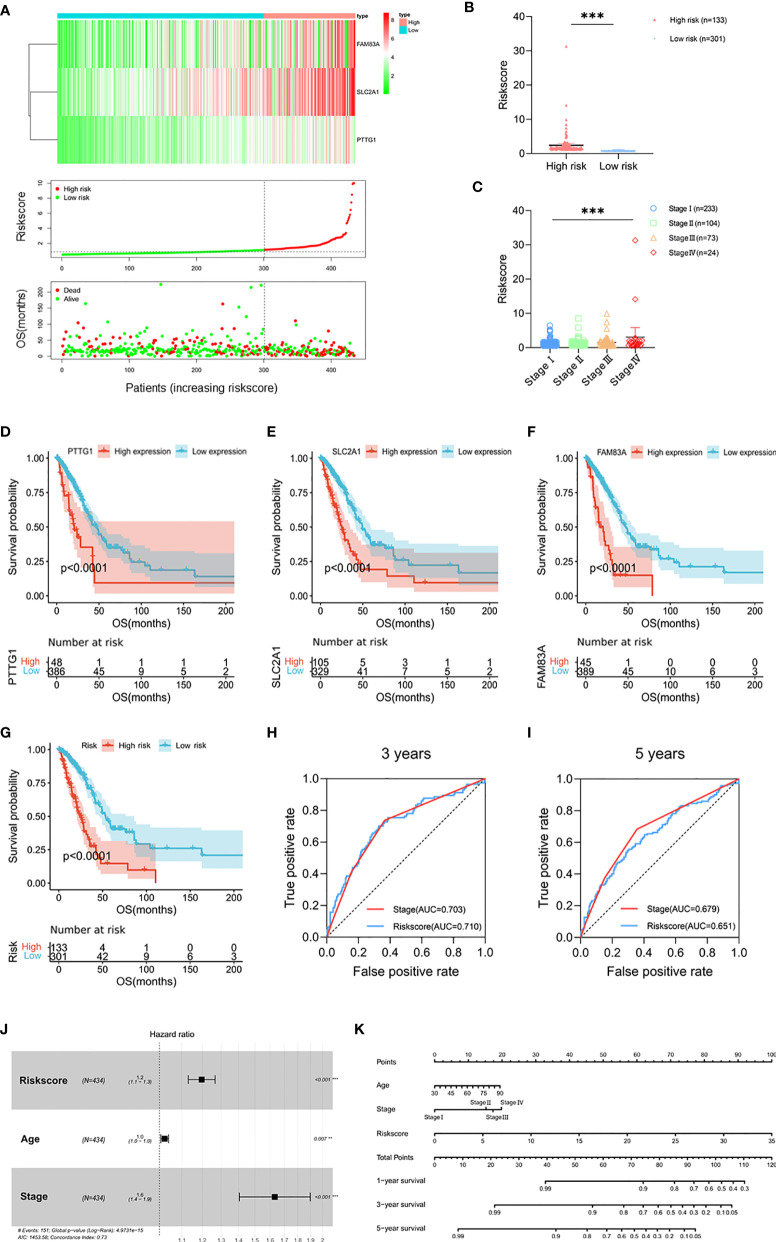
Prognostic risk model built by *PTTG1*, *SLC2A1*, and *FAM83A*. **(A)** Heat map of *PTTG1*, *SLC2A1*, and *FAM83A* in the TCGA cohort (upper figure). Distribution of risk score (middle figure), and OS (lower figure). **(B)** The risk score in the high-risk group (n = 133) and low risk group (n = 301). **(C)** Distribution of risk scores classified by stage. **(D, G)** Kaplan–Meier curves for OS by expression of **(D)** *PTTG1*, **(E)** *SLC2A1*, **(F)** *FAM83A*, and **(G)** risk subgroups in the TCGA cohort. Significance was determined using the log-rank p test. **(H, I)** Time-dependent ROC curves measuring the predictive value of the risk score and stage at **(H)** 3 years and **(I)** 5 years in the TCGA cohort. **(J)** Forest plot showing the independent prognostic factors for OS in the TCGA cohort (multivariate Cox regression analysis). **(K)** Nomogram for predicting 1-, 3-, and 5-year overall survival in the TCGA cohort. Mean with 95% CI; **p < 0.01, and ***p < 0.001.

In order to simplify the clustering of molecular subtypes, we built a prognostic risk model based on the signatures of molecular subtypes and evaluated it in terms of both prognostic value and immunological characteristics. We performed univariate Cox, Lasso, and multivariate Cox regression analysis based on 275 DEGs (**Supplementary Figures 5A–C**). Three genes, *PTTG1*, *SLC2A1*, and *FAM83A*, were screened out as signatures of molecular subtypes (p < 0.05). According to the expression of signatures and the prognosis information, we calculated the risk score of each patient (risk score = *PTTG1* expression level* 0.026624 + *SLC2A1* expression level* 0.005851 + *FAM83A* expression level* 0.006776) and divided the patients into high-risk group (n = 133) and low-risk group (n = 301) according to the optimal cutoff value (**Figures 3A, B** and **Supplementary Table 5**). The risk scores of stage IV patients were higher than those of stage I patients, which tended to increase with stage (p < 0.001; **Figure 3C**). Survival analysis showed that in the TCGA cohort, the high expression groups of signatures had poor prognosis (p < 0.0001; **Figures 3D–F**), and the survival time of patients in the high-risk group was shorter than that in the low-risk group (p < 0.0001; **Figure 3G**). Next, the prognostic value of the risk score was evaluated. ROC analysis showed that the prediction ability of risk score at the 3-year survival status was superior to that of the clinical stage (AUC = 0.710 versus AUC = 0.703, p < 0.001), while it was weaker than that of the clinical stage at 5 years (AUC = 0.651 versus AUC = 0.679, p < 0.001; **Figures 3H, I**). Multivariate Cox regression analysis showed that the risk score was independent and significantly correlated with prognosis (p < 0.001; **Figure 3J**). In addition, we constructed a nomogram consisting of independent prognostic factors; there were great OS rates at 1, 3, and 5 years for younger patients, lower stage, and lower risk score (**Figure 3K**).

In addition, we compared the distribution of CNV and SNP between risk subgroups and found that there was no significant difference in CNV and SNP of *TP53*/*KRAS* aberrations between risk subgroups (**Supplementary Figures 5D, E**). Furthermore, the gene mutation frequency of the DNA damage repair (DDR) pathway was low. There was no DDR pathway gene in the 15 genes with the most significant SNP, and the mutation rate of PTEN with the lowest mutation frequency was only 2%. Gene Set Enrichment Analysis (GSEA) showed that cell cycle (NES = 2.28, FDR < 0.001) and P53 signaling pathway (NES = 1.99, FDR = 0.009) were correlated with the high-risk group (**Supplementary Figures 5F, G**). These findings were similar to the previous KEGG analysis, suggesting that the heterogeneity of the mRNA expression between the risk subgroups may be an important factor affecting the progression.

### Validation of the Prognostic Risk Model in the LUAD Cohort

**Figure 4:**
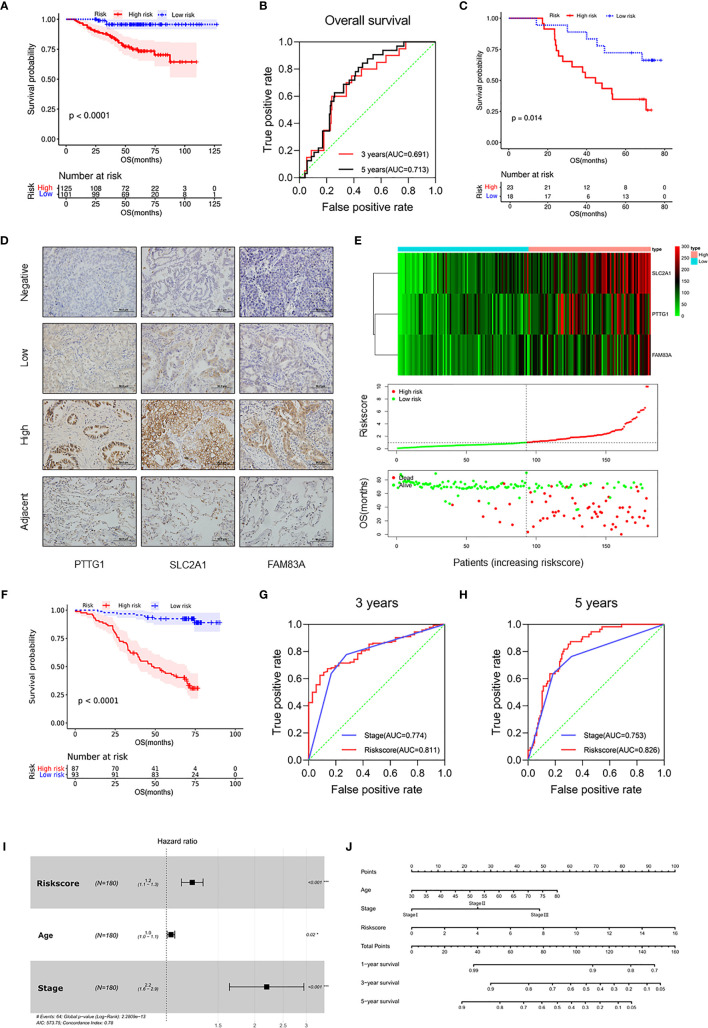
Validation of the prognostic risk model in the LUAD cohort. **(A)** Kaplan–Meier curves for OS by risk subgroups in the GSE31210. **(B)** Time-dependent ROC curves measuring the prognostic value of the risk score at 3 and 5 years in the GSE31210. **(C)** Kaplan–Meier curves for OS by risk subgroups in the mRNA level from the Tianjin cohort. **(D)** Representative IHC images for staining of signatures in 180 samples of patients with LUAD (Olympus, ×400 magnification, bar = 50 μm). **(E)** Heat map of *PTTG1*, *SLC2A1*, and *FAM83A* in the Tianjin cohort (upper figure). Distribution of risk score (middle figure), and OS (lower figure). **(F)** Kaplan–Meier curves for OS by risk subgroups in the Tianjin cohort. **(G, H)** Time-dependent ROC curves measuring the prognostic value of the risk score and stage at **(G)** 3 years and **(H)** 5 years in the Tianjin cohort. **(I)** Forest plot showing the independent prognostic factors for OS in the Tianjin cohort (multivariate Cox regression analysis). **(J)** Nomogram for predicting 1-, 3-, and 5-year overall survival in the Tianjin cohort. *p < 0.05 and ***p < 0.001.

Survival analysis from the external dataset (n = 226) showed significant differences in clinical outcomes between subgroups (p < 0.001), consistent with the TCGA cohort (**Figure 4A** and **Supplementary Table 6**). The ROC analysis showed that AUC values of the risk score for 3 years and 5 years OS were 0.691 and 0.713, respectively (**Figure 4B**). In addition, RT-qPCR was used to verify the predictive ability of the model at the mRNA level in the Tianjin cohort (n = 41), and there were significant differences in survival between subgroups (p = 0.014; **Figure 4C** and **Supplementary Table 7**). ROC analysis showed that the risk score was a better predictor of prognosis than TNM at 3 years (AUC = 0.733 versus AUC = 0.659, p < 0.001) and 5 years (AUC = 0.705 versus AUC = 0.702, p < 0.001; **Supplementary Figures 6A, B**).

In order to further validate the prognostic value of the prognostic risk model at the protein level, 180 samples of LUAD patients from the Tianjin Cancer Hospital were enrolled in this study (**Table 1** and **Supplementary Table 8**). Immunohistochemical staining of *PTTG1*, *SLC2A1*, and *FAM83A* was performed on the paraffin-embedded continuous sections (**Figure 4D**). The survival analysis showed that OS was significantly longer in the low expression group compared with the high expression group (p < 0.0001; **Supplementary Figures 6C–E**). Next, we recalculated the risk score for each patient at the protein level (risk score = *PTTG1* expression level * 0.005561 + *SLC2A1* expression level * 0.008432 + *FAM83A* expression level * 0.008010) and divided the patients into the high-risk group (n = 87) and low-risk group (n = 93; **Figure 4E**). The OS (**Figure 4F**) of the high-risk group was significantly worse than that of the low-risk group (p < 0.0001; **Table 2**). The distribution of risk scores increased with clinical stage, and patients with advanced tumor were more at risk (p < 0.01; **Supplementary Figures 6F, G**). Further, ROC analysis showed that risk score was a better predictor of prognosis than clinical stage at 3 years (AUC = 0.811 versus AUC = 0.774, p < 0.001) and 5 years (AUC = 0.826 versus AUC = 0.753, p < 0.001) in the Tianjin cohort (**Figures 4G, H**). Multivariate Cox regression analysis showed that risk score, age, and clinical stage of patients also were independent prognostic factors in this cohort (p < 0.001; **Figure 4I**). Moreover, the nomogram listed the three independent prognostic factors, and OS rates at 1, 3, and 5 years can be effectively predicted based on the scores related to each variable (**Figure 4J**). In conclusion, the prognostic value of the prognostic risk model built by *PTTG1*, *SLC2A1*, and *FAM83A* was credible.

**Table 1:** Clinical parameters and their association with the risk score in the Tianjin cohort.

Clinical parameters		N (%)	PTTG1		p-value	SLC2A1		p-value	FAM83A		p-value	Risk score		p-value
			Low	High		Low	High		Low	High		Low	High	
Age (years)														
	<65	132 (73.3)	58	74	0.305	59	73	0.892	95	37	0.673	71	61	0.345
	≥65	48 (26.7)	17	31		22	26		33	15		22	26	
Gender														
	Males	79 (43.9)	33	46	0.980	30	49	0.094	49	30	0.017*	35	44	0.080
	Females	101 (56.1)	42	59		51	50		79	22		58	43	
Clinical stage														
	I	98 (54.4)	45	53	0.128	59	39	<0.0001***	82	16	<0.0001***	65	33	<0.0001***
	II	24 (13.3)	12	12		8	16		14	10		9	15	
	III	58 (32.3)	18	40		14	44		32	26		19	39	
T classification														
	T1	95 (52.8)	43	52	0.632	57	38	<0.0001***	74	21	**0.118**	60	35	0.001***
	T2	63 (35.0)	24	39		20	43		38	25		28	35	
	T3	16 (8.9)	5	11		2	14		12	4		2	14	
	T4	6 (3.3)	3	3		2	4		4	2		3	3	
N classification														
	N0	113 (62.8)	51	62	0.227	63	50	0.001***	92	21	<0.0001***	68	45	0.003**
	N1	14 (7.8)	7	7		5	9		9	5		8	6	
	N2	53 (29.4)	17	36		13	40		27	26		17	36	
Smoke														
	No smoking	114 (63.3)	49	65	0.638	61	53	0.003**	93	21	<0.0001***	65	49	0.059
	Smoking	66 (36.7)	26	40		20	46		35	31		28	38	

*p < 0.05, **p < 0.01, and ***p < 0.001.

Bold values indicate statistical significance p < 0.05.

**Table 2:** Univariate survival analysis of clinical parameters and the risk score with PFS and OS in the Tianjin cohort.

Clinical parameters		n	PFS	p-value	OS	p-value
			Hazard ratio		Hazard ratio	
			(95% CI)		(95% CI)	
Age (years)						
	<65	132	1.299	0.227	1.336	0.282
	≥65	48	(0.825~2.046)		(0.760~2.348)	
Gender						
	Males	79	0.846	0.403	0.732	0.212
	Females	101	(0.568~1.261)		(0.443~1.208)	
Clinical stage						
	I+II	122	3.304	<0.0001***	4.834	<0.0001***
	III	58	(2.067~5.281)		(2.710~8.623)	
Tumor						
	T1	95	2.300	<0.0001***	2.408	0.0005***
	T2+T3+T4	85	(1.536~3.444)		(1.460~3.971)	
Lymph node						
	N0	113	2.954	<0.0001***	4.387	<0.0001***
	N1+N2	67	(1.913~4.562)		(2.557~7.527)	
Smoke						
	No smoking	114	1.091	0.675	1.364	0.221
	Smoking	66	(0.721~1.652)		(0.810~2.298)	
PTTG1						
	Low	75	3.936	<0.0001***	3.798	<0.0001***
	High	105	(2.654~5.837)		(2.316~6.228)	
SLC2A1						
	Low	81	4.105	<0.0001***	8.024	<0.0001***
	High	99	(2.758~6.110)		(4.890~13.17)	
FAM83A						
	Low	128	2.943	<0.0001***	3.909	<0.0001***
	High	52	(1.809~4.785)		(2.175~7.025)	
Risk score						
	Low	93	5.519	<0.0001***	10.96	<0.0001***
	High	87	(3.638~8.372)		(6.597~18.20)	

***p < 0.001.

Bold values indicate statistical significance p < 0.05.

### Comparison of TIME Between Risk Subgroups

**Figure 5:**
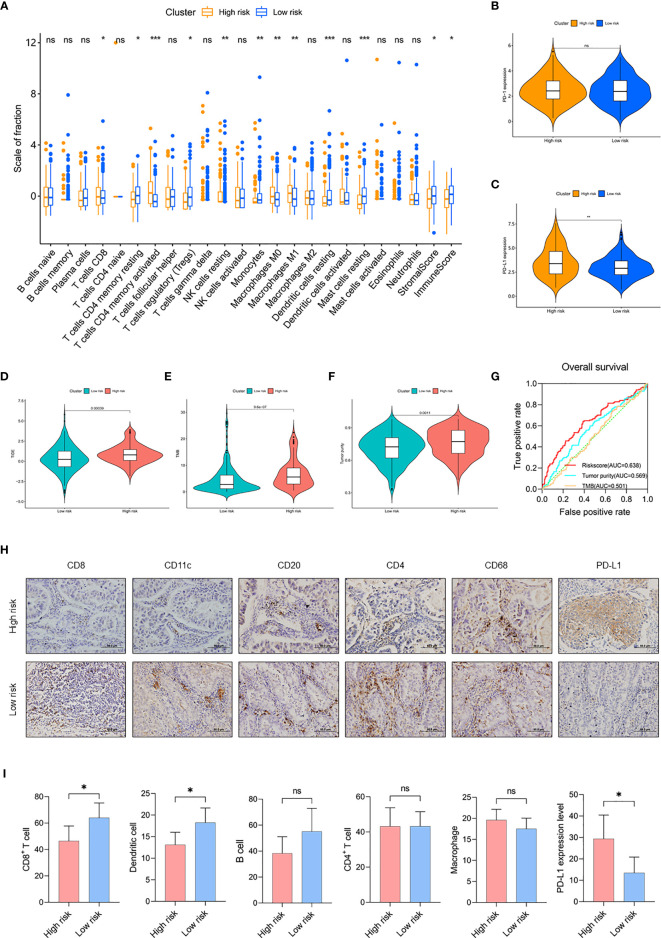
Comparison of TIME between risk subgroups. **(A)** The fractions of 22 immune cell types, stromal score, and immune score in the high-risk group (n = 133) and low-risk group (n = 301) of the TCGA cohort. **(B, C)** Expression level of PD-1 **(B)** and PD-L1 **(C)** in risk subgroups of the TCGA cohort. **(D–F)** Value of **(D)** TIDE, **(E)** TMB, and **(F)** tumor purity in risk subgroups of the TCGA cohort. **(G)** Time-dependent ROC curves measuring the predictive value of the risk score, TMB, and tumor purity in the TCGA cohort. **(H)** Representative IHC images for staining of CD8+ T cell, CD11c+ DC, CD20+ B cell, CD4+ T cell, CD68+ macrophage, and PD-L1 classified by risk score in 100 samples of LUAD patients from Tianjin (Olympus, ×400 magnification, bar = 50 μm). **(I)** The counts of CD8+ T cell, DC, B cell, CD4+ T cell, and macrophage and the expression level of PD-L1 in the Tianjin cohort. High-risk group (n = 52) and low risk group (n = 48). Mean with 95% CI; *p < 0.05, **p < 0.01, and ***p < 0.001. ns, not statistically significant.

Next, we evaluated the level of immune cell infiltration in the risk subgroups of the TCGA cohort. In the low-risk group, the total immune score, the stromal score, and the fraction of DC were higher (p < 0.05; **Figure 5A**). In addition, the fractions of CD8+ T cells and activated CD4+ T cells were lower (p < 0.05), and both the fractions of resting CD4+ T cells and Tregs were higher (p < 0.05) in the low-risk group. For macrophage, the fractions of M0 and M1 were lower (p < 0.01) in the low-risk group, while there was no difference in M2 between the two subgroups. Then, we detected the expression of immune checkpoints. The results showed that the expression level of PD-L1 in the high-risk group was significantly higher than in the low-risk group (p < 0.01), but there was no difference in PD-1 and other immune checkpoints (**Figures 5B, C** and **Supplementary Figures 7A–D**). TIDE analysis showed that the low-risk group may have better immunotherapy responses, and the risk score was correlated with the TIDE score (**Figure 5D** and **Supplementary Figure 7E**). These findings suggested that the risk subgroups could partially represent the immunological characteristics of the molecular subtypes.

In addition, we compared and analyzed the association between risk score and TMB/tumor purity. We found higher TMB and tumor purity in the high-risk group (p < 0.01), the risk score was correlated with TMB and tumor purity (p < 0.01), and the risk score had better prognostic ability than TMB and tumor purity (risk score AUC = 0.638, tumor purity AUC = 0.569, TMB AUC = 0.501), suggesting the potent utility of this model for clinical individualized treatment (**Figures 5E–G** and **Supplementary Figures 7F, G**).

To validate the differences of TIME between the risk subgroups, we detected the expression of immune cell markers and microenvironment in LUAD samples. In this study, staining of CD8+ T cells (CD8), DC (CD11c), B cells (CD20), CD4+ T cells (CD4), and TAM (CD68) was used to show the infiltrating landscape of immune cells, which accounted for the largest five immune cell proportions in the TIME (**Figure 5H** and **Supplementary Table 9**). The results showed that CD8+ T cells infiltrated less in the high-risk group (p < 0.05), and the risk score was negatively correlated with CD8+ T cell counts (r = -0.2387, p = 0.0435); DC also infiltrated less in the high-risk group (p < 0.05), and the risk score was negatively correlated with DC counts (r = -0.2630, p = 0.0290); the expression of PD-L1 was higher in the high-risk group and the risk score was positively correlated with the expression of PD-L1 (r = 0.2427, p = 0.0359). In addition, there was no difference in the infiltration of B cells, CD4+ T cells, and macrophage between the risk subgroups (**Figure 5I** and **Supplementary Figure 7H**). Due to the limited samples in the LUAD cohort, the patterns of immune cell infiltration could not completely correspond to the TCGA cohort. In conclusion, the risk subgroups showed significantly different TIME, a high-risk group with an immunosuppressive microenvironment expressed a high level of PD-L1, and the low-risk group with an immunoreactive microenvironment had a high level of immune cell infiltration.

## Discussion

Genome instability is clearly an enabling characteristic that is causally associated with the acquisition of hallmark capabilities; some clonal expansions may well be triggered by non-mutational changes affecting the regulation of gene expression (27). Genomics and epigenetics work together to regulate gene expression; cells undergo further genetic diversification that enables tumor progression, relapse, and resistance to therapy (28). Recent advances have revealed intratumoral heterogeneity in cell states, epigenetic profiles, and interactions with the tumor microenvironment, and these axes of potentially heritable intratumoral variation may provide additional cues for cancer evolution (29). Thus, the integration of multiple layers of information for individual cancer cells can therefore help identify new mechanisms underlying and clinically relevant definitions for tumor heterogeneity, candidate treatment targets, and tumor biomarkers (30).

**Figure 6:**
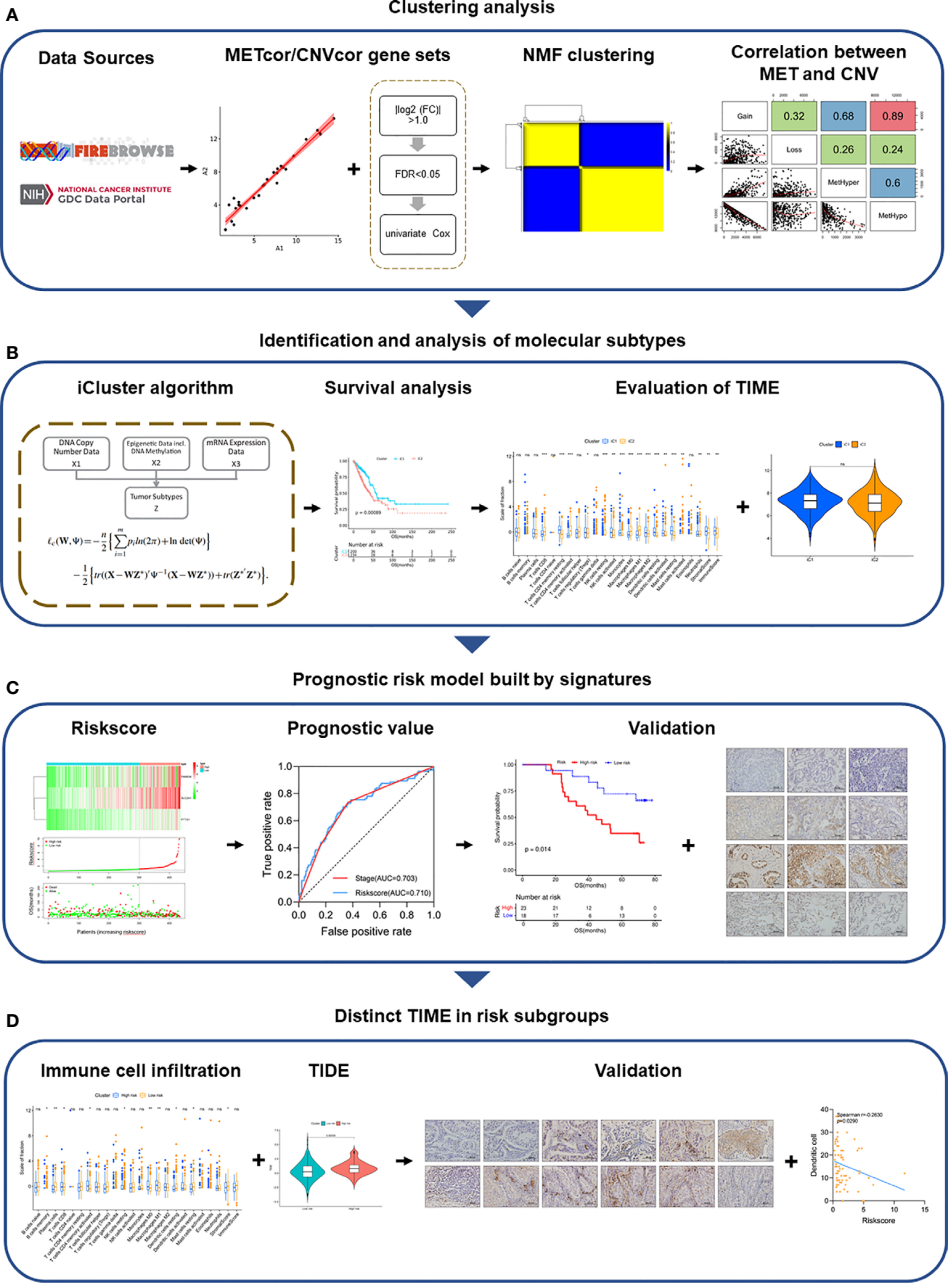
Overview of the study design. **(A)** Clustering analysis of METcor/CNVcor gene sets by NMF. **(B)** Identification and analysis of molecular subtypes. **(C)** Signatures of molecular subtypes built the prognostic risk model, which was validated in multiple cohorts. **(D)** Distinct TIME of risk subgroups was validated by IHC.

In this study, we integrated the MET, CNV, and mRNA expression data in 443 samples with LUAD from the TCGA project, confirming the dysregulation of mRNA expression caused by MET and CNV aberrations (**Figure 6**). Furthermore, we found that the *TP53* mutation rate (54%) was the highest in the cohort. *TP53* is a driver gene of LUAD revealed by large-scale genomic studies, and *TP53* is one of the most common somatic mutations regulating cell cycle and apoptosis (31, 32). Amplification of *TP53* made p53 activation upon transcriptionally downregulated genes for many central cell cycle proteins which achieved cell cycle arrest (33). Enrichment analysis of DEGs showed that the P53 signaling, the cell cycle, and apoptosis pathways were enriched, suggesting that tumor cells of iC2 may have stronger proliferation and anti-apoptotic ability. The results further revealed the potential mechanism of the heterogeneity between iC1 and iC2. We hypothesized that the P53 signaling pathway may be involved in the regulation of the biological function of heterogeneous tumors between the two subtypes of LUAD.

Subsequently, we found differences in immune characteristics between subtypes. Professional killer cells include NK and CD8+ T cells, which represent some of the most effective immune defense mechanisms against cancer cells; stromal CD8+ T cell density has independent prognostic impact in resected NSCLC and is a good candidate marker (34, 35). Tregs can suppress the activation of the immune system, maintain immune tolerance to self-antigens, and contribute to immunosuppression of antitumor immunity, which is critical for tumor immune evasion in epithelial malignancies, including lung cancer (36). Accumulating evidence suggests that PD-1/PD-L1-targeting antibodies are effective for treating many types of human cancer including NSCLC (37, 38). However, the status of PD-L1 expression on tumor cells alone is not sufficient to identify patients who might respond to PD-1/PD-L1 blockade immunotherapy; mismatch repair and the presence of tumor-infiltrating lymphocytes including CD8+ T cell and Tregs in tumor samples could also influence the immune response (39, 40). TIDE was a computational method used to model two primary mechanisms of tumor immune evasion: the induction of T cell dysfunction in tumors with high infiltration of cytotoxic T lymphocytes (CTL) and the prevention of T cell infiltration in tumors with a low CTL level (41). Compared with single detection of the PD-L1 expression level or infiltration of CD8+T cells, TIDE is more comprehensive to evaluate the response of patients to immunotherapy. With the lower TIDE score, immunotherapy of immune checkpoint inhibitors (ICIs) may be beneficial to iC1.

In order to screen out personality signatures that could represent heterogeneous molecular subtypes, DEGs in the mRNA expression profile between the two subtypes were processed by Cox and Lasso algorithms. Finally, *PTTG1*, *SLC2A1*, and *FAM83A* were selected as signatures to build a prognostic model. Physiological *PTTG1* properties include securin activity and DNA damage/repair regulation (42, 43). Tumorigenic mechanisms for *PTTG1* action involve cell transformation and aneuploidy, apoptosis, and tumorigenic microenvironment feedback (44). The main function of *SLC2A1* is to supply the cell with glucose by facilitated diffusion of glucose molecules across the plasma membrane, which is a key protein in the cellular energy metabolism pathway and could mediate metabolic deregulation (45, 46). A stable metabolism was required for T cell differentiation and effector function; metabolic deregulation could cause T cell dysfunction (47, 48). *FAM83A* as MET signature shows a significant correlation to tumor mutation burden and DNA damage response pathways (49, 50). DNA repair deficiency can trigger a more robust and long-lasting immune response, and strong TIL infiltration with tumor eradication with the presence of frameshift-mutated neoantigens, mutational landscape, and mismatch-repair deficiency are related to the immune response as candidate biomarkers for ICI immunotherapy (51, 52). The three genes *PTTG1*, *SLC2A1*, and *FAM83A* have different functions, and the prognostic risk model established by complementary advantages integrated their respective characteristics. The expression of signatures led to individual differences in prognostic risk. In order to provide a quantitative method for clinicians to predict the prognosis of LUAD patients, we constructed a nomogram using independent prognostic risk factors including risk scores. The nomogram generates an individual probability of a clinical event by integrating diverse prognostic and determinant variables and meets our desire for clinically integrated models and fulfill our drive toward personalized medicine (53).

Interestingly, the differences in immune cell infiltration between the high-risk group and low-risk group were very similar to the differences between the two molecular subtypes. The total immune score and the fraction of DC were higher in the low-risk group compared with the high-risk group. More recently, as a novel definition of cancer, immune scores implicated at all stages a complex and dynamic interaction between tumor cells and the immune system, allowing TIME to be used to represent immune parameters related to patient survival (54). DC play key roles in the initiation and regulation of innate and adaptive immune responses and influence immunity and tolerance in cancer settings, and there is currently much interest in modulating DC function to improve cancer immunotherapy (55, 56). Circulating monocytes give rise to mature macrophages and are also heterogeneous themselves (57). In this study, we detected CD68 as an indicator of TAM, including M1 and M2 macrophages, which are difficult to characterize due to their heterogeneity (58). In fact, we also stained the infiltration of NK cells (CD56) in the TIME, but since NK cells were mainly distributed in the peripheral blood, we could not detect their infiltration. Moreover, the risk score was correlated with TIDE score, TMB, and tumor purity, suggesting that the high-risk group may be associated with T cell dysfunction, higher TMB, and higher tumor purity. Immune cell infiltration caused low purity and high TMB with neoantigens, which can bring long-lasting clinical benefits. However, there are limitations for using TMB to identify potential patients; the response rate in patients with tumors that have TMB-H (≥20 mutations/Mb) is only 45% (59, 60). These findings indicated that the risk score is strongly associated with immune cell infiltration; a high-risk score indicates poor prognosis and poor immune cell infiltration. The patients in the low-risk group have a lower expression of PD-L1, infiltrating more immune cells. Therefore, immunotherapy targeted at PD-L1 may be beneficial to patients in the low-risk group.

In conclusion, we found an integrative perspective to identify subtypes; signatures of subtypes may be useful indicators for predicting prognosis, and patients in the low-risk group may benefit more from immunotherapy, thus facilitating personalized management in LUAD.

## Data Availability Statement

The original contributions presented in the study are included in the article/**Supplementary Material**. Further inquiries can be directed to the corresponding author.

## Ethics Statement

The study involving samples were reviewed and approved by the Tianjin Medical University Cancer Institute and Hospital ethics committee. The patients provided their written informed consent to participate in this study.

## Author Contributions

ZH, BL, and YG performed the experiments. LW and FK coordinated the patient sample acquisition and ethical approval of the study. LY, ZH, and BL conceived the study and conducted the data analyses. All authors contributed to the article and approved the submitted version.

## Funding

This work was supported by grants from the National Key Technology R&D Program (No. 2018YFC1313400); the National Natural Science Foundation of China (No. 81974246); and the Tianjin Research Innovation Project for Postgraduate Students (No. 2020YJSB164).

## Conflict of Interest

The authors declare that the research was conducted in the absence of any commercial or financial relationships that could be construed as a potential conflict of interest.

## Publisher’s Note

All claims expressed in this article are solely those of the authors and do not necessarily represent those of their affiliated organizations, or those of the publisher, the editors and the reviewers. Any product that may be evaluated in this article, or claim that may be made by its manufacturer, is not guaranteed or endorsed by the publisher.
