# Supplementary material for: Signatures of Multi-Omics Reveal Distinct Tumor Immune Microenvironment Contributing to Immunotherapy in Lung Adenocarcinoma
Source: Front Immunol. 2021 Sep 3;12:723172. doi: 10.3389/fimmu.2021.723172 (PMC8446514; doi:10.3389/fimmu.2021.723172)
Supplement: Supplementary file 1 [file DataSheet_1.pdf]

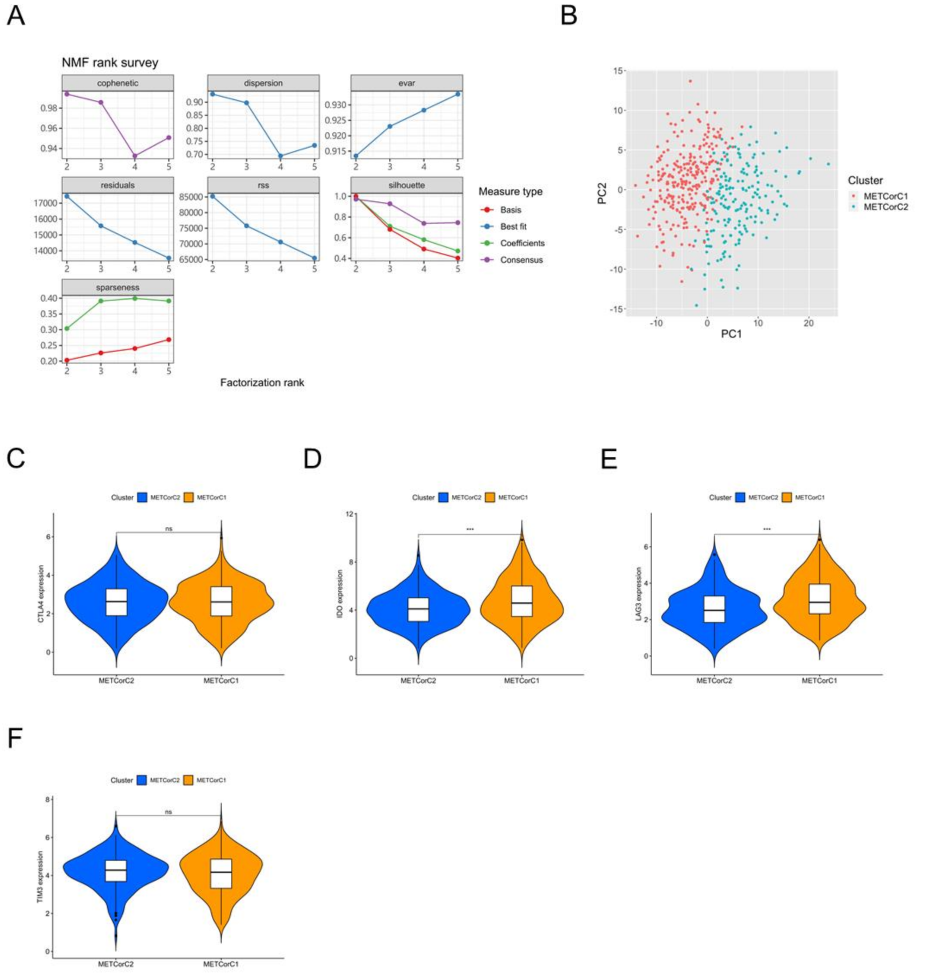

Supplementary Figure 1. (A) The process of clustering METcor clusters with NMF. (B) The PCA of the two METcor clusters. The red dots represent the patients in METCorC1 (n=267). The green dots represent the patients in METCorC2 (n=176). (C-F) Expression level of (C) CTLA4, (D) IDO, (E) LAG3 and (F) TIM3 in METCorC1 and METCorC2. *$p < 0.05$, **$p < 0.01$ and ***$p < 0.001$.

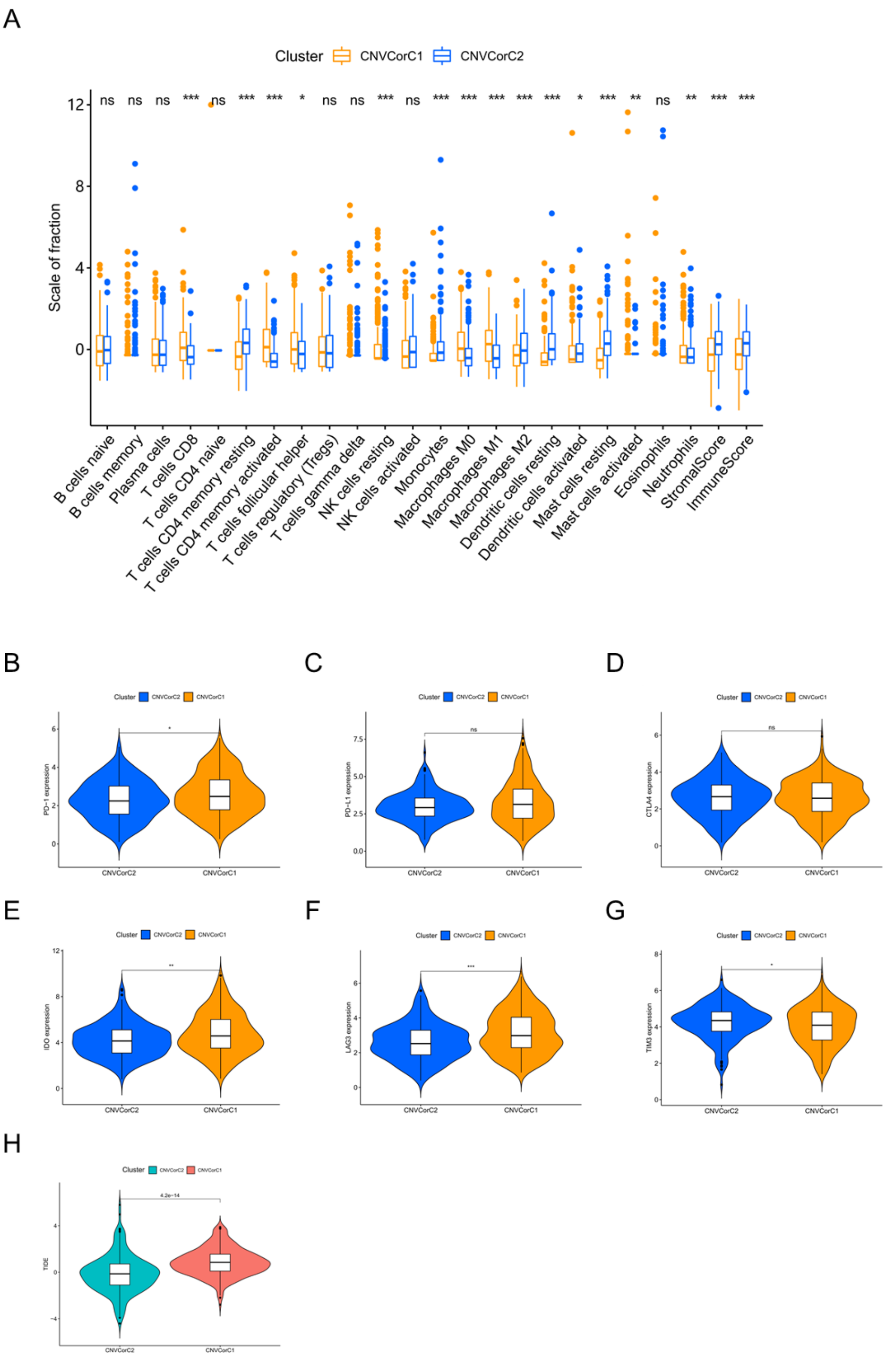

Supplementary Figure 2. (A) The fractions of 22 immune cell types, stromal score and immune score in CNVcor clusters. (B-G) Expression of (B) PD-1, (C) PD-L1, (D) CTLA4, (E) IDO, (F) LAG3 and (G) TIM3 in CNVcor clusters. (H) TIDE score in CNVcor clusters. CNVCorC1 (n=251) and CNVCorC2 (n=192). *p < 0.05, **p < 0.01 and ***p < 0.001.

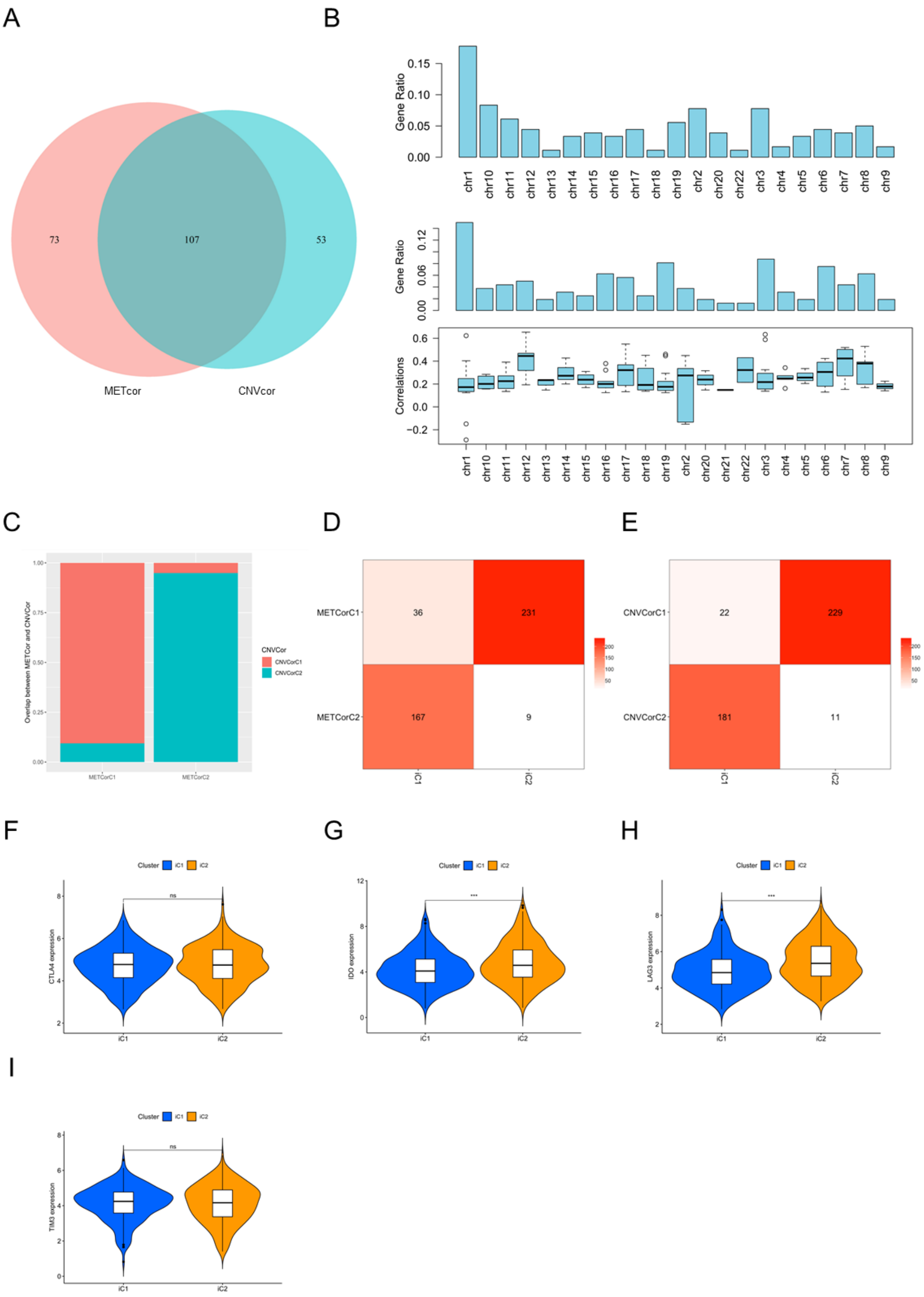

Supplementary Figure 3. (A) Venn diagram of overlapping METcor genes and CNVcor genes. (B) Box plot of METcor genes chromosomal distribution (upper figure). Box plot of CNVcor genes chromosomal distribution (middle figure) and correlations (lower figure). (C) The overlap between METcor and CNVcor clusters. (D) The correlation of METcor clusters and iC subtypes. (E) The correlation of CNVcor clusters and iC subtypes. (F-I) Expression level of (F) CTLA4, (G) IDO, (H) LAG3 and (I) TIM3 in iC subtypes. $*p < 0.05$, $**p < 0.01$ and $***p < 0.001$.

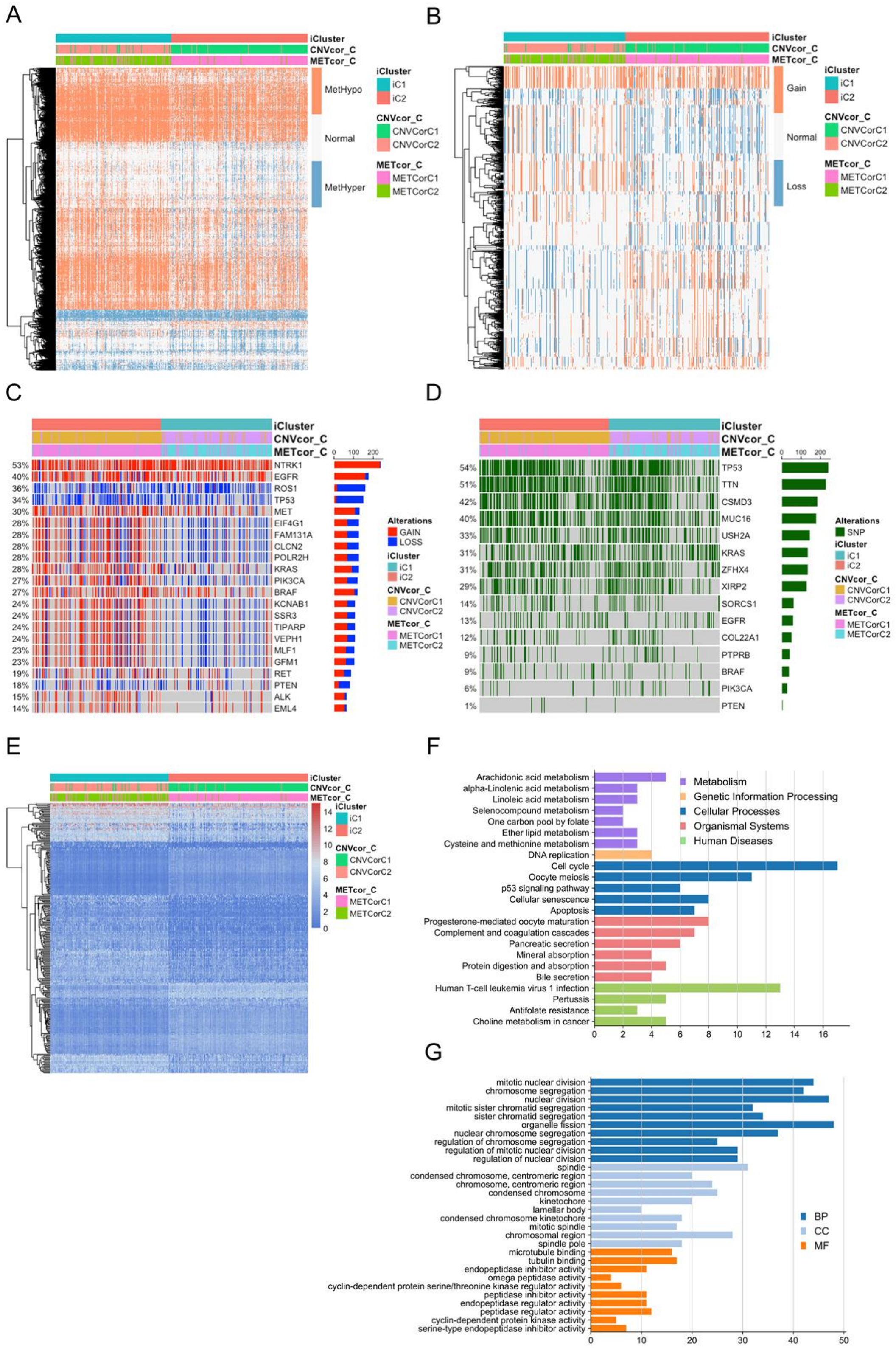

Supplementary Figure 4. (A) MET and (B) CNV distribution in iC1 and iC2. (C) CNV distribution of 22 driver genes and (D) SNP distribution of top 15 significant genes in iC1 and iC2. (E) Heatmap of DEGs in mRNA expression level between iC1 and iC2. (F) KEGG enrichment and (G) GO functional annotation analysis of 275 DEGs between iC1 and iC2; $p < 0.05$.

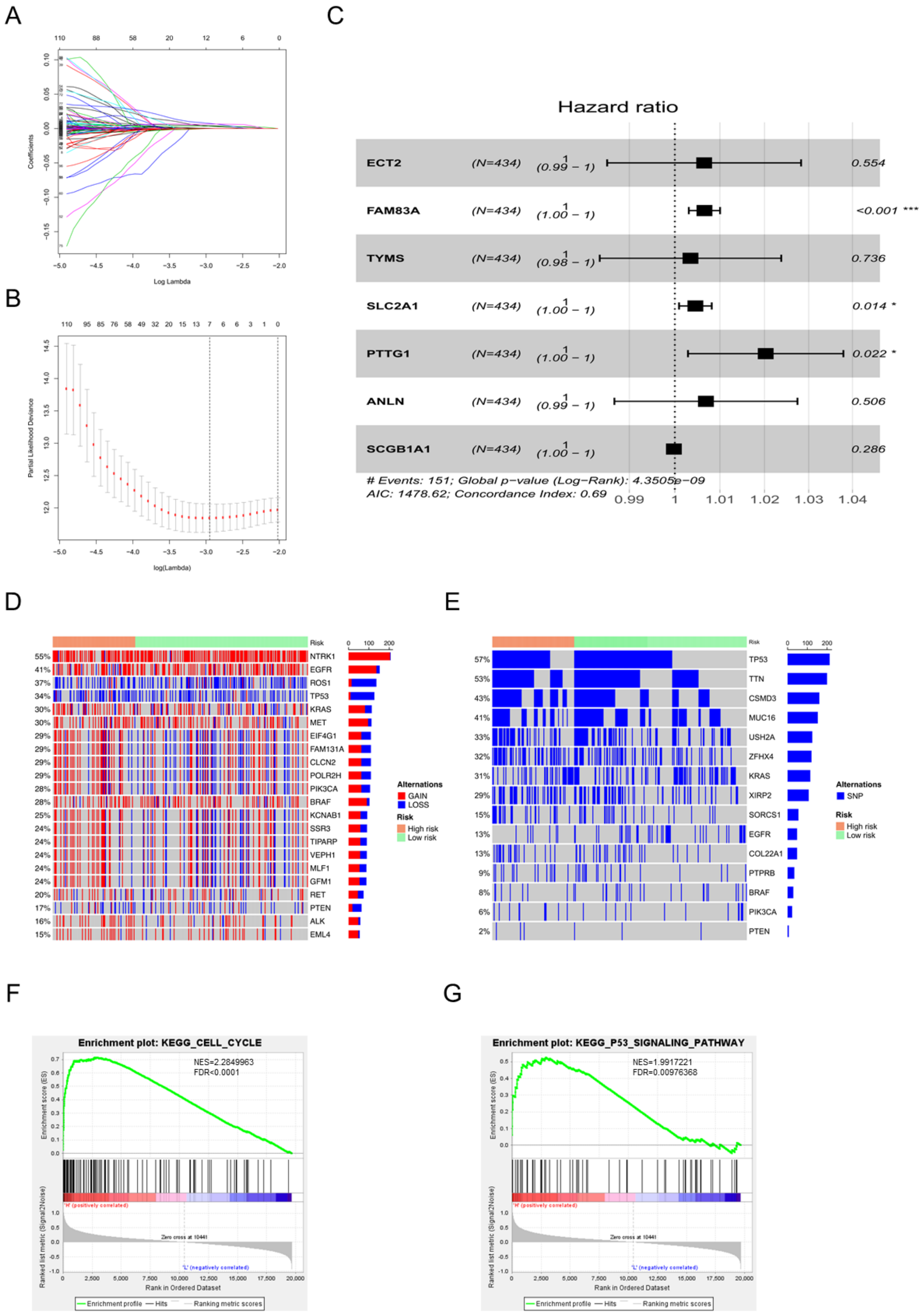

Supplementary Figure 5. (A-B) Lasso regression analysis and (C) multivariate Cox regression analysis were performed to determine signatures. (D) CNV distribution of 22 driver genes and (E) SNP distribution of top 15 significant genes in high risk group and low risk group. Enrichment analysis showed (F) cell cycle and (G) P53 signaling pathways were significantly upregulated in the high risk group. *p < 0.05, **p < 0.01 and ***p < 0.001.

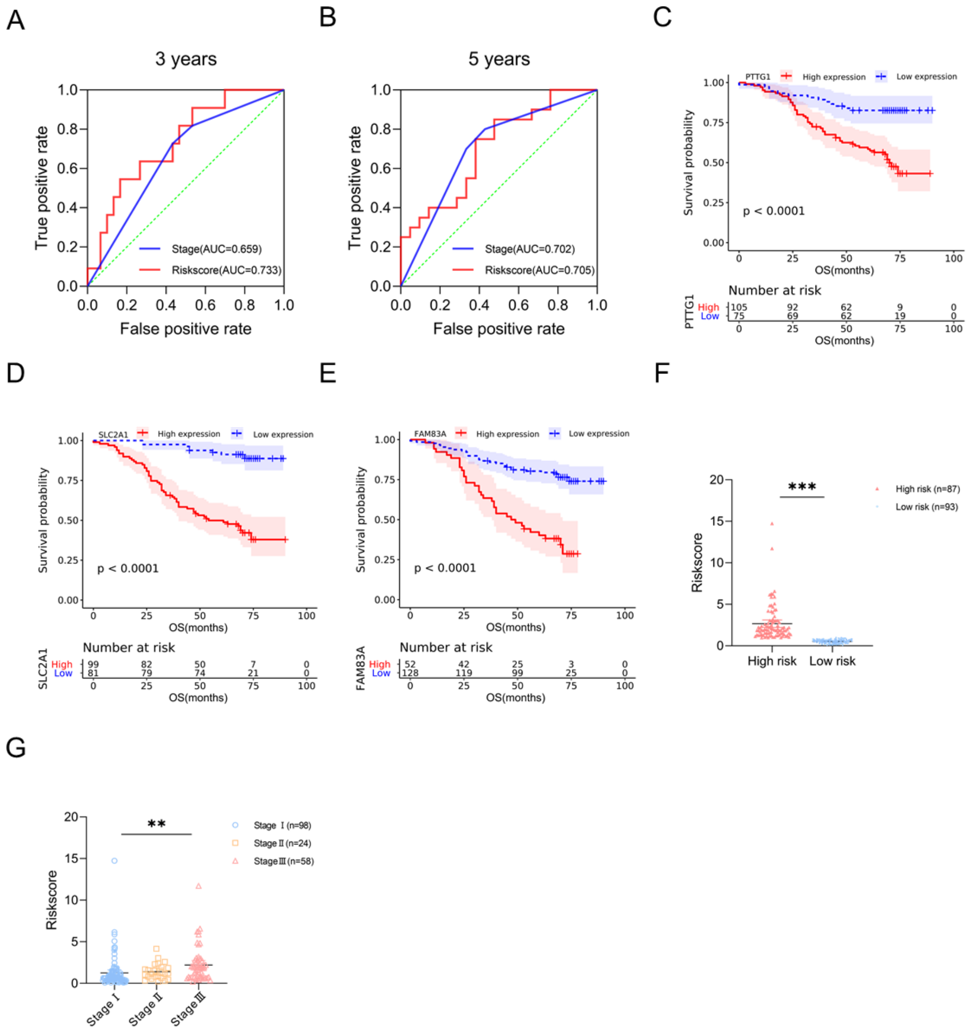

Supplementary Figure 6. (A-B) Time-dependent ROC curves measuring the predictive value of the risk score and stage at (A) 3 years and (B) 5 years in the mRNA level from Tianjin cohort. (C-E) Kaplan-Meier curves for OS by expression of (C) PTTG1, (D) SLC2A1 and (E) FAM83A in the Tianjin cohort. Significance was determined using log rank p test. (F) Distribution of risk score in risk subgroups. (G) Distribution of risk score classified by stage. Mean with 95% CI; *p < 0.05, **p < 0.01 and ***p < 0.001.

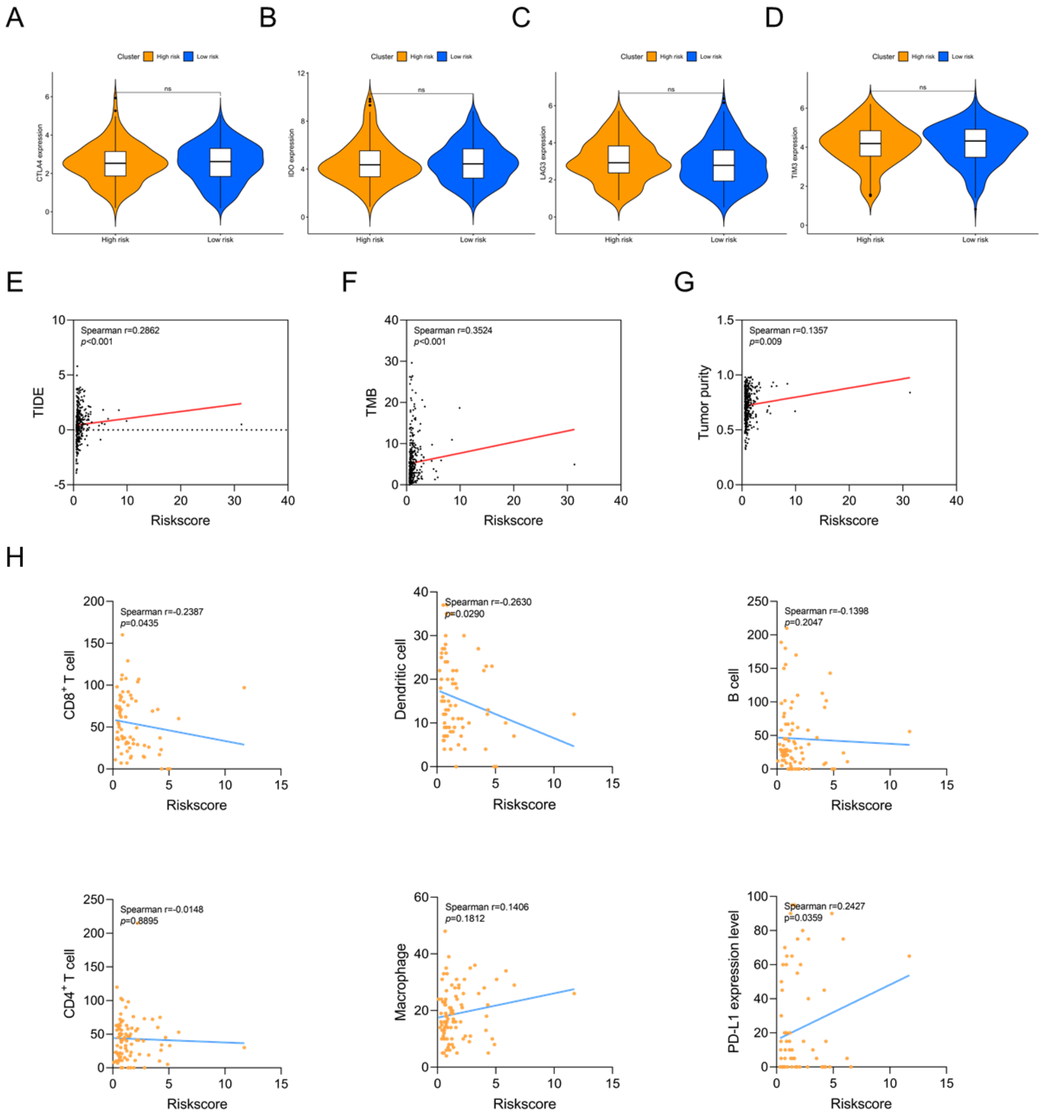

Supplementary Figure 7. (A-D) Expression level of (A) CTLA4, (B) IDO, (C) LAG3 and (D) TIM3 in risk subgroups. (E-G) The distribution of (E) TIDE, (F) TMB and (G) tumor purity with the risk score. (H) The distribution of CD8+ T cell, DC, B cell, CD4+ T cell, macrophage counts and the expression level of PD-L1 with the risk score.
